# Securing genetic integrity in freshwater pearl mussel propagation and captive breeding

**DOI:** 10.1038/s41598-021-95614-2

**Published:** 2021-08-06

**Authors:** Juergen Geist, Helmut Bayerl, Bernhard C. Stoeckle, Ralph Kuehn

**Affiliations:** 1 Aquatic Systems Biology Unit, Department of Life Science Systems, Technical University of Munich, 85354 Freising, Germany; 2 Molecular Zoology Unit, Department of Molecular Life Sciences, Technical University of Munich, 85354 Freising, Germany; 3 Department of Fish, Wildlife and Conservation Ecology, New Mexico State University, 2980 South Espina, Box 30003, Las Cruces, NM 88003-8003 USA

**Keywords:** Genetics, Zoology, Ecology, Limnology, Ecology, Biodiversity, Ecological genetics, Freshwater ecology

## Abstract

Securing genetic integrity is of key importance in conservation-oriented captive breeding programs releasing juveniles into the wild. This is particularly true for species such as the endangered freshwater pearl mussel (*Margaritifera margaritifera*) for which a number of captive breeding facilities has been established in Europe. The core objective of this study was to compare the genetic constitution of 29 cohorts of captive-bred freshwater pearl mussels from five different breeding facilities in Austria, France, Luxembourg and Germany, with their original 14 source populations from nine major European drainages, based on microsatellite markers. Captive-bred mussels represented 11 different genetic clusters, suggesting an important contribution of the breeding stations to securing the genetic diversity of the species. In almost all cases, the cultured offspring closely resembled the genetic constitution of the source mussels as revealed from the STRUCTURE analysis and the generally high assignment of offspring to the original source populations. The majority of captive-bred cohorts had an increased inbreeding coefficient and decreased genetic variability compared to their source populations as measured by A_R_ and H_O_. Highest numbers of deformed juveniles coincided with very low levels of H_O_ < 0.05. Since erosion of genetic diversity in captive breeding was mostly evident in individual year-cohorts, long-term breeding over multiple years can minimize such effects. The systematic selection of priority populations for conservation, effective breeding strategies avoiding effects of in- and outbreeding by genetically informed selection of parent individuals, and a network of collaboration among the different breeding facilities would be very useful to increase resilience and effectiveness.

## Introduction

Freshwater biodiversity is globally in decline, with freshwater mussels being among the most affected taxonomic groups^1,2^. One of the species that receives greatest attention is the freshwater pearl mussel (*Margaritifera margaritifera*) which simultaneously fulfills the criteria of indicator, keystone, flagship and umbrella species^3^. Freshwater pearl mussels can reach ages of more than 100 years and have an exceptional life cycle that involves a parasitic life stage on a fish host that can either be Atlantic salmon (*Salmo salar*), brown trout (*Salmo trutta*), or both in European populations^4,5^. Degradation of pearl mussel habitats, mostly related to siltation and colmation of interstitial spaces in the stream bed where juvenile pearl mussels burrow, have resulted in a lack of recruitment and severe declines of many pearl mussel populations throughout their European range^6,7^. Since restoration of stream beds requires consideration of entire catchments and the restoration of natural flow dynamics^8^, this approach is time-consuming and costly. At the same time, many pearl mussel populations are overaged and expected to die out in the near future. Since the first descriptions of the ongoing threats and population declines^9^, the downward trend of populations has even become worse^7^. This development has prompted the creation of a first European CEN standard for a single species^10^ as well as several captive breeding efforts in different countries^11–14^. Captive breeding of freshwater pearl mussel was first established in the Czech Republic by Jaroslav Hruška^11^ and is mostly based on induced release of glochidia larvae from parent mussels, a subsequent infestation and holding of fish hosts as well as collection and raising of dropped-off juveniles^14^.

Due to the strong conservation interest in freshwater pearl mussel, its genetic constitution has been studied in depth both throughout its European^15–22^ as well as its North American^23^ distributions, facilitating the consideration of priority populations for conservation in captive breeding. A wise selection of parents or an annual replacement of breeders can help secure the genetic diversity in captive breeding efforts. Still, there are multiple factors that may compromise the genetic constitution of captive-bred mussels compared to their parent populations. For instance, the selection of small numbers of parent mussels that are not representative for the genetic constitution of the entire population can already narrow the basis of genetic variability within the breeding program. During infestation of host fish, the compatibility of the used species and strains among mussel and fish as well as the age of the fish all can have effects on attachment rates, survival and growth^5,24,25^. Physiology of the host fish immune system related to temperature^26^, the effects of different loading densities of mussel larvae on swimming performance of the host^27^ as well as the duration of the parasitic phase^28,29^ and associated nutrient transfer from the host to the mussel^30^ all have a potential selective effect on the mussels during the parasitic phase. In the subsequent post-parasitic (i.e. juvenile) phase, stock origin and environmental conditions^31^, food quantity as well as water and sediment quality^32–34^, but also changing of culture systems and the cleaning regimes^35^ were found to affect survival and growth. Generally, mortalities are highest until the juveniles reach the first millimeter^36^.

In order to make the most of recent advances in freshwater mussel propagation and restoration^37^, consideration of genetic effects during the breeding process and objective evaluation of ongoing breeding activities is needed yet still lacking. In addition to the technical optimization of breeding facilities for freshwater mussels^38^, such information can be useful in identifying the most suitable captive breeding techniques for retaining a maximum of the genetic-evolutionary potential of mussels^3^. The core objective of this study thus was to analyze the genetic constitution of captive-bred freshwater pearl mussels from different European breeding facilities and compare their genetic diversity and differentiation with the original populations. These served as a source for the parent mussels and captive-bred juveniles have been, or are intended to be released into the same streams. Specifically, we hypothesized that (1) breeding efforts would be suitable to secure the genetic identity of the captive bred mussels as indicated by low genetic differentiation between original populations and captive-bred juveniles, (2) there would be no decrease of the genetic variability in pearl mussel during the captive breeding as evident from highly similar genetic diversity indices such as H_o_ and A_R_ between original populations and captive-bred juveniles.

## Material and methods

### Study design

Our study design was based on the comparison of population genetic parameters of natural freshwater pearl mussel populations and corresponding cohorts of juveniles propagated at specialized breeding stations. We included five facilities from four European countries (Germany, Austria, Luxembourg, France) in our sampling regime. In these, freshwater pearl mussels were propagated from a total of 14 different source populations representing nine main drainage systems: Elbe (Weiße Elster, Wolfsbach/Zinnbach), Danube (Kleine Ohe, Naarn, Wolfertsrieder Bach), Rhine (Sûre), Maas (Rulles, Anlier), Loire (Le Sarthon), Orne (La Rouvre), Sienne (L’Airou), Blavet (Le Bonne Chère, Le Loc’h) and Aulne (L’Elez) (Table 1). Source Populations of the streams Sûre, Rulles and Anlier were located on Belgian territory and juveniles were reared in Luxembourg. For genetic analyses we used data of 382 adult specimens from respective source populations collected between 2003 and 2019^15–17^ in their natural habitats (based on representative sample collection over multiple locations per stream) or directly from individuals of the broodstock held at the breeding station as practiced in Austria^39,40^. Genetic constitution of those mussels was compared to results of 897 propagated juveniles comprising 29 different cohorts which were selected from different age classes ranging from 0 + to ca. 16 years. They were collected either while being held at the breeding facility, or from gravel-filled cage-boxes submerged in the streams of intended release, or from already released and marked individuals.

**Table 1:** Population characteristics and population genetic summary statistics of *Margaritifera margaritifera* source populations and corresponding cohorts of propagated juveniles.

Drainage	Co	Population	Type	Pop. size	Code	N	Age	Def	Loci (fixed L.)	MLG	A	A_R_	H_E_	H_O_	F_ST_ (O-S)	S_(FST)_	F_IS_	F_ind_	F	P_(HW)_
Elbe	D	Weiße Elster	S	< 50 (2004)	WE-S	6	–	–	9 (2)	6	2.6	2.5	0.436	0.278	–		0.388	0.435	0.04	n.s
	D	Weiße Elster	O	–	WE-O1	50	14–16 +	2	9 (2)	50	3.8	2.5	0.399	0.339	0.090	***	0.152	0.336	0.23	***
	D	Weiße Elster	O	–	WE-O2	40	11–14 +	1	9 (2)	39	4.0	2.5	0.406	0.304	0.058	**	0.253	0.386	0.12	***
	D	Wolfsbach/Zinnbach	S	2000 (2015)	WZ-S	50	–	–	9 (3)	44	2.9	2.0	0.331	0.312	–		0.058	0.329	0.18	n.s
	D	Wolfsbach/Zinnbach	O	–	WZ-O1	50	8–16 +		9 (3)	49	3.2	2.2	0.379	0.347	0.062	***	0.085	0.310	0.12	n.s
	D	Wolfsbach/Zinnbach	O	–	WZ-O2	29	0 +	0	9 (1)	28	3.4	2.4	0.422	0.446	0.055	***	− 0.058	0.254	0.11	n.s
	D	Wolfsbach/Zinnbach	O	–	WZ-O3	31	0 +	0	9 (2)	31	2.8	2.3	0.400	0.364	0.043	***	0.090	0.310	0.12	***
Danube	D	Kleine Ohe	S	1300 (2012)	KO-S	32	–	–	9 (1)	32	2.9	2.3	0.424	0.369	–		0.131	0.318	0.22	n.s
	D	Kleine Ohe	O	–	KO-O	25	12 +	0	9 (1)	25	3.8	2.7	0.504	0.321	0.146	***	0.368	0.407	0.16	***
	D	Wolfertsrieder Bach	S	800 (2008)	WB-S	21			9 (1)	21	4.0	2.9	0.531	0.460			0.135	0.298	0.10	n.s
	D	Wolfertsrieder Bach	O		WB-O	20	8		9 (1)	20	3.4	2.6	0.457	0.401	0.088	**	0.125	0.311	0.22	n.s
	A	Naarn	S	50 (2017)	NA-S	30	–	–	9 (4)	13	1.8	1.5	0.155	0.030	–		0.811	0.627	0.18	***
	A	Naarn	O	–	NA-O1	20	2 +	–	9 (6)	6	1.3	1.3	0.094	0.033	− 0.004	n.s	0.650	0.597	0.17	***
	A	Naarn	O	–	NA-O2	36	0 +	–	9 (5)	9	1.6	1.3	0.083	0.009	0.021	n.s	0.890	0.652	0.31	***
Rhine	B	Sûre	S	200 (2004)	SU-S	26	–	–	9 (6)	8	1.3	1.2	0.082	0.038	–		0.536	0.584	0.14	**
	B	Sûre	O	–	SU-O	30	0 +	0	9 (1)	23	3.4	2.0	0.290	0.173	0.264	***	0.358	0.430	0.04	***
Maas	B	Rulles	S	200 (2004)	RU–S	25	–	–	9 (8)	3	1.1	1.1	0.052	0.044	–		0.152	0.532	0.31	n.d
	B	Rulles	O	–	RU–O	30	0 +	2	9 (2)	26	2.6	2.0	0.293	0.176	0.297	***	0.398	0.435	0.01	***
	B	Anlier	S	1000 (2004)	AN-S	26	–	–	9 (6)	12	1.7	1.4	0.107	0.062	–		0.429	0.536	0.20	***
	B	Anlier	O	–	AN-O	30	3 +	0	9 (4)	23	2.0	1.7	0.205	0.113	0.345	***	0.455	0.499	0.19	***
Loire	F	Le Sarthon	S	152 (2006)	ST-S	26	–	–	9 (5)	7	1.6	1.3	0.073	0.017	–		0.766	0.625	0.17	***
	F	Le Sarthon	O	–	ST-O1	30	3 +	18	9 (7)	3	1.2	1.1	0.034	0.000	0.638	***	1.000	0.666	0.24	***
	F	Le Sarthon	O	–	ST-O2	27	0 +	7	9 (7)	4	1.3	1.2	0.038	0.016	0.584	***	0.573	0.618	0.00	**
Orne	F	La Rouvre	S	110 (2002)	RO-S	16	–	–	9 (3)	9	1.9	1.5	0.123	0.035	–		0.725	0.596	0.00	***
	F	La Rouvre	O	–	RO-O1	30	2 +	26	9 (6)	4	1.4	1.1	0.029	0.000	0.035	n.s	1.000	0.664	0.27	***
	F	La Rouvre	O	–	RO-O2	30	1 +	10	9 (5)	6	1.4	1.4	0.171	0.000	0.120	n.s	1.000	0.703	0.19	***
	F	La Rouvre	O	–	RO-O3	30	2 +	2	9 (7)	3	1.2	1.1	0.015	0.007	0.052	n.s	0.496	0.641	0.13	n.s
Sienne	F	L’ Airou	S	59 (2007)	AI-S	18	–	–	9 (3)	11	2.0	1.6	0.174	0.049	–		0.723	0.600	0.06	***
	F	L’ Airou	O	–	AI-O	30	1 +	1	9 (6)	5	1.3	1.3	0.111	0.000	0.379	***	1.000	0.681	0.09	***
Blavet	F	Le Bonne Chère	S	962 (2009)	BC-S	25	–	–	9 (6)	7	1.6	1.4	0.095	0.062	–		0.349	0.503	0.19	***
	F	Le Bonne Chère	O	–	BC-O1	30	4 +	0	9 (6)	8	1.4	1.3	0.094	0.048	0.086	n.s	0.491	0.549	0.25	***
	F	Le Bonne Chère	O	–	BC-O2	29	2 +	0	9 (7)	4	1.3	1.1	0.025	0.011	0.462	***	0.551	0.630	0.18	n.d
	F	Le Bonne Chère	O	–	BC-O3	30	1 +	1	9 (7)	4	1.3	1.1	0.028	0.007	0.407	***	0.736	0.644	0.26	***
	F	Le Bonne Chère	O	–	BC-O4	30	0 +	1	9 (6)	14	1.6	1.4	0.111	0.050	0.362	***	0.555	0.548	0.21	***
	F	Le Loc ‘ h	S	180 (2008)	LO-S	48	–	–	9 (4)	23	1.9	1.5	0.164	0.069	–		0.620	0.570	0.24	***
	F	Le Loc ‘ h	O	–	LO-O1	30	4 +	2	9 (5)	6	1.7	1.5	0.159	0.004	0.120	***	0.977	0.688	0.06	***
	F	Le Loc ‘ h	O	–	LO-O2	30	3 +	19	9 (5)	14	1.8	1.6	0.179	0.044	0.298	***	0.755	0.601	0.19	***
	F	Le Loc ‘ h	O	–	LO-O3	30	1 +	0	9 (7)	4	1.3	1.3	0.107	0.000	0.085	***	1.000	0.679	0.23	***
	F	Le Loc ‘ h	O	–	LO-O4	30	0 +	0	9 (6)	5	1.3	1.2	0.044	0.004	0.272	***	0.918	0.658	0.21	***
Aulne	F	L’ Elez	S	400 (2005)	EL-S	54	–	–	9 (6)	6	1.4	1.1	0.018	0.006	–		0.400	0.646	0.35	n.d
	F	L’ Elez	O	–	EL-O1	30	5 +	3	9 (6)	5	1.3	1.1	0.021	0.015	0.014	n.s	0.314	0.630	0.15	n.d
	F	L’ Elez	O	–	EL-O2	30	4 +	1	9 (7)	5	1.3	1.2	0.056	0.015	0.070	n.s	0.739	0.634	0.19	***
	F	L’ Elez	O	–	EL-O3	30	3 +	0	9 (7)	5	1.4	1.2	0.045	0.007	0.040	n.s	0.836	0.645	0.19	***

Drainage, country of source population (Co.; D = Germany, A = Austria, B = Belgium, F = France), population (waterbody), population type (S = source population, O = offspring cohort), estimated population size (year of estimation), population code, sample size (N), age of propagated mussels at the time of genetic sampling, number of morphologically deformed specimens in the sample (Def.), number of genotyped microsatellite loci and number of fixed loci, number of distinct multilocus genotypes (MLG), mean number of alleles per locus (A), mean allelic richness per population (A_R_), expected and observed heterozygosity (H_E_, H_O_), F_ST_-value between offspring cohorts and corresponding parent populations (F_ST_ (O-P)) and respective *p*-values (P(_FST)_), inbreeding coefficient (F_IS_), individual inbreeding coefficient (F_ind_), F-value based on the 2MOD program (F) and results of Hardy–Weinberg probability tests for deviation from expected Hardy–Weinberg proportions (P_(HW)_). Significance levels of all tests after sequential Bonferroni correction^41^: *** *P* ≤ 0.001, ** *P* ≤ 0.01, * *P* ≤ 0.05, n.s. = not significant, n.d. = not defined.

### Sampling and DNA extraction

We collected 40–100 µL haemolymph from the foot tissue of adult individuals and juveniles exceeding 30 mm total shell length by following the methodology of Geist & Kuehn^16^. This sampling technique is not harmful for the mussels which were returned alive to their original sites immediately after sampling. Juveniles of cohorts between 0 + and 5 years of age and a total shell length between < 1 mm and ca. 1 cm were sampled using whole individuals due to their small size. Obvious deformations of juvenile mussels (e.g., irregular and compressed shell shapes and growth patterns) were additionally noted. Haemolymph was transferred into 1.7 mL Eppendorf tubes while juveniles were kept alive in containers filled with water. Both were cooled at 4 °C and subsequently transported to the laboratories of the Technical University of Munich, Germany. Samples were prepared for DNA extraction by centrifuging haemolymph at 14.000 g for 5 min and discarding the supernatant whereas juveniles were transferred to individual reaction tubes. To ensure efficient lysis of the tissue, the smallest individuals (~ 1 mm) were crushed inside the reaction tube using a metal probe, or one shell valve with adhering tissue was used. Genomic DNA was then isolated from cellular pellets and juveniles using the NucleoSpin Tissue Kit (Macherey–Nagel GmbH, Düren, Germany) according to the manufacturer’s protocol for tissue samples and eluted in 80 µL of BE buffer.

### PCR and genotyping

DNA samples were genotyped at nine standard species-specific microsatellite markers (MarMa2671, MarMa3050, MarMa3621, MarMa4143, MarMa4322, MarMa4726, MarMa5167, MarMa5280 and MarMa5023) as described by Geist et al.^15^ and Geist & Kuehn^16,17^. Polymerase chain reactions (PCRs) were performed in a total volume of 12.5 µL containing 25 ng genomic DNA, 0.2 µM of each primer, 0.2 mM of each dNTP, 3 mM MgCl_2_ for eight Loci (2 mM MgCl_2_ for Locus 5280), 1 × FirePol® PCR buffer BD (0.8 M Tris–HCl, 0.2 M (NH_4_)SO_4_ and 0.5 U FirePol® Taq DNA polymerase (Solis Biodyne, Tartu, Estonia) under the cycling conditions described in Geist et al.^15^ and Geist & Kuehn^16^. Forward primers were end-labelled with Cy5 fluorescent dye and PCR products were separated on 5% denaturing 19:1 acrylamid:bisacrylamid gels on an ALFexpressII DNA analyser (Amersham Pharmacia Biotech) and allele lengths were scored using ALLELELINKS 1.02 software. To ensure consistent allele scoring between individual lanes and among gels, two internal size standards were included per lane^42^ as well as 11 size standards and one previously genotyped reference sample in two separate lanes.

### Statistical analyses

Microsatellite allele frequencies, the mean number of alleles per locus (A), allelic richness (A_R_) as a standardized measure of the number of alleles corrected for sample size, expected and observed heterzygosities (H_E_, H_O_) and inbreeding coefficient (F_IS_) were calculated using Fstat v. 2.9.3^43^. We used Genepop v. 4.7.3^44^ to test genotypic distributions for conformance with Hardy–Weinberg expectations using the probability test^45^, to calculate pairwise F_ST_ values^46^ between source populations and offspring cohorts and to estimate the significance of genotypic differentiation between these populations pairs. All probability tests were based on the Markov chain^47,48^ method using 10,000 dememorization steps, 100 batches and 5000 iterations per batch. The number of distinct multilocus genotypes (MLG) was determined using the R-package Poppr v. 2.8.3^49^. The R-package Adegenet v. 2.1.1^50^ was used to determine mean individual inbreeding coefficients (F_ind_) for each source population and offspring cohort by calculating for each individual the probability of being homozygous at a locus: p(h) = F + (1 − F) $\sum\nolimits_{i} {p_{i}^{2} }$ and summing up log-likelihoods over all microsatellite loci to account for multilocus genotypes, where F refers to the probability of an individual to inherit two identical alleles from a single ancestor and *p*_*i*_ refers to the frequency of allele *i* in a population. Additionally, we estimated relatedness between individuals within populations based on the F-value of the 2mod program^51^ which provides information on the probability that two genes share a common ancestor within a population and is correlated with effective population size. The Markov chain Monte Carlo (MCMC) simulation was run for 200,000 iterations and the initial 10% of the data were discarded to avoid dependence on starting conditions. We used Structure v. 2.3.4 software^52^ to determine and visualize the number of genetic clusters (K) present among source populations and to assign probabilities of cluster memberships to propagated individuals. We tested values of K ranging from 2 to 20 under the admixture model and assuming correlated allele frequencies using 20,000 burn-ins, 200,000 MCMC repetitions and 10 iterations per K to assess the convergence of ln P(X│K). The software package Clumpak v. 1.1^53^ was used to infer the most likely number of K based on the ΔK method of Evanno et al.^54^. The implemented program Clumpp v. 1.1^55^ was used to find the optimal individual alignments of replicated cluster analyses using the LargeKGreedy algorithm and 2000 random input orders which were then visualized using Distruct v. 1.1^56^. Bayesian clustering techniques may produce biased results in terms of cluster recognition when working with unbalanced sample sizes^57,58^. We therefore validated Structure results using the multivariate approach of discriminant analysis of principal components (DAPC)^59^ implemented in the software package Adegenet v. 2.1.1^50^ in R v. 3.6.2^60^ which is less sensitive to uneven sampling^58^. DAPC first transforms the data using principal component analysis (PCA) and then performs a Discriminant Analysis (DA) on the retained principal components (PC). We retained 13 PCs which explained 85% of the total variation of the data set. Results of the DAPC were visualized by assigning the first three PCs to intensities of the color channels of the RGB system. Similar generated colors thus correspond to similar genetic constitutions of respective individuals or populations^59^.

## Results

### Genetic integrity

**Figure 1:**
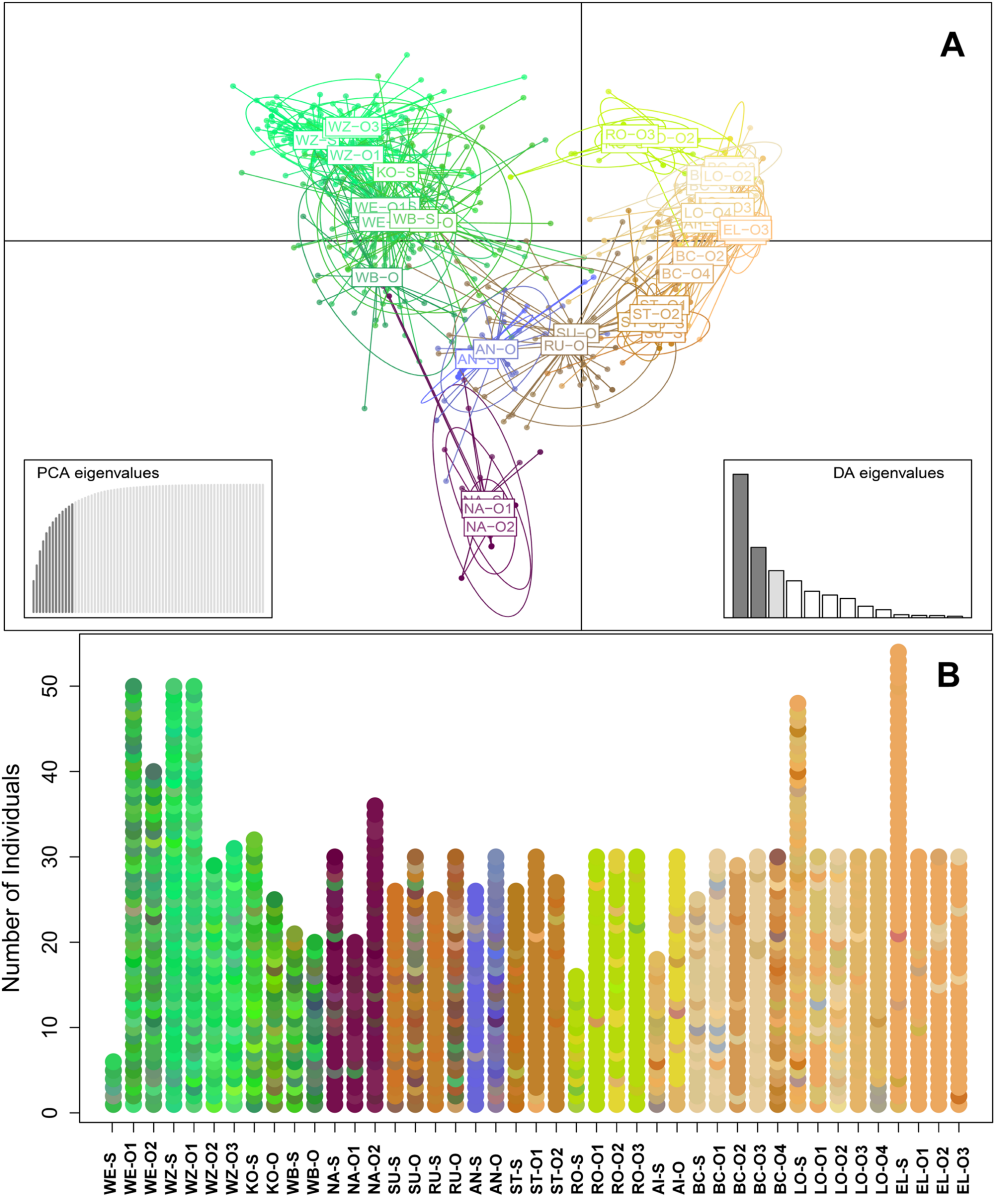
(**A**) Clustering of 1300 individuals of 14 *Margaritifera margaritifera* source populations and 29 corresponding cohorts of propagated juveniles based on discriminant analysis of principal components (DAPC) using the first 13 principal components and 3 discriminant functions. Individuals are depicted as dots, populations are represented by inertia ellipses and mean population color based on the DAPC; population codes according to Table 1. (**B**) Individual genetic constitution of 1300 *Margaritifera margaritifera* from 14 source populations and respective cohorts of propagated juveniles based on discriminant analysis of principal components (DAPC) using the first 13 principal components and 3 discriminant functions. The color of the dots corresponds to the results of the DAPC with similar colors indicating similar genetic constitution.

The captive breeding efforts over the five investigated rearing facilities were found to represent a diversity of genetically differentiated clusters of freshwater pearl mussel. Overall, eleven genetic clusters were identified as the most probable outcome using the ΔK method (Table 2, Fig. S2). In almost all cases, the cultured offspring closely resembled the genetic constitution of the source mussels as revealed from the Structure analysis and the generally high assignment of offspring to the original source populations. In 13 out of 29 cases (45%), a 100% assignment to the original population was found, and in 25 out of the 29 cases (86%), more than 80% of individuals were correctly assigned (Table 2). Otherwise, low levels of assignment occurred in populations (BC, LO, RU and SU) that generally had extremely low values of genetic variability (Table 1) and where drift effects due to the limited availability of a small number of gravid broodstock mussels for the breeding were expected. The result of the DAPC is consistent with the findings of the Structure analyses and graphically illustrates the genetic differentiation between source and captive-bred mussels based on color codes of multilocus genotype frequencies of each individual sampled (Fig. 1).

**Table 2:** Assignment of 1300 *Margaritifera margaritifera* individuals to genetic clusters identified based on genotyping nine polymorphic microsatellite markers and individual membership coefficients derived from Structure and Clumpp software.

Facility	Code	N	Genetic cluster											A [%]
			1	2	3	4	5	6	7	8	9	10	11	
1	WE-S	6		*1*		*1*					*4*			
	WE-O1	50		**7**							**43**			100.0
	WE-O2	40		**9**							**31**			100.0
1	WZ-S	50		*49*							*1*			
	WZ-O1	50		**49**							**1**			100.0
	WZ-O2	29		**28**							**1**			100.0
	WZ-O3	31		**31**										100.0
2	KO-S	32									*32*			
	KO-O	25									**24**	1		96.0
2	WB-S	21									*21*			
	WB-O	20		2							**18**			85.7
	NA-S	30											*30*	
3	NA-O1	20											**20**	100.0
	NA-O2	36											**36**	100.0
4	SU-S	26							*5*			*21*		
	SU-O	30	1			6			**1**	1	1	**18**	2	63.3
4	RU–S	25										25		
	RU–O	30		1				2	13	2	3	**3**	6	10.0
4	AN-S	26				*26*								
	AN-O	30				**28**		1		1				93.3
5	ST-S	26	*1*									*25*		
	ST-O1	30						2		1		**27**		90.0
	ST-O2	27								1		**26**		96.3
5	RO-S	16	*2*				*13*			*1*				
	RO-O1	30			1		**29**							96.7
	RO-O2	30	**12**				**17**			**1**				100.0
	RO-O3	30					**30**							100.0
5	AI-S	18	*4*						*2*	*11*		*1*		
	AI-O	30	**22**							**8**				100.0
5	BC-S	25			*25*									
	BC-O1	30			29			1						96.7
	BC-O2	29			29									100.0
	BC-O3	30			30									100.0
	BC-O4	30			**18**			1	2	2		7		60.0
5	LO-S	48	*16*					*11*	*2*	*18*		*1*		
	LO-O1	30			4			**13**		**13**				86.7
	LO-O2	30	**2**		21			**4**		**3**				30.0
	LO-O3	30			1			**11**		**18**				96.7
	LO-O4	30	**3**							**26**		**1**		100.0
5	EL-S	54						*54*						
	EL-O1	30						**28**	1	1				93.3
	EL-O2	30			5			**25**						83.3
	EL-O3	30			3			**26**				1		86.7

Individuals were derived from 14 source populations (S) and corresponding offspring cohorts (O) propagated at five different European breeding facilities. Genetic clusters present in source populations are highlighted in italic. Bold numbers refer to the number of samples in offspring cohorts belonging to the same genetic cluster(s) as the corresponding source population, regular numbers refer to the number of samples belonging to a genetic cluster not represented in the source population. Dashed lines delimit corresponding parental populations and offspring cohorts. N = number of analyzed samples, A = percentage of propagated individuals assigned to a genetic cluster represented in the corresponding source population.

Most F_ST_ values between captive-bred mussels with their original populations from the wild were low to moderate, indicating high similarity in the alleles present and their frequencies in both groups (Table 1). This was for instance obvious with the Austrian NA-O1 and NA-O2 compared to NA-S, a culturing system where the parent mussels are permanently maintained in a flow-through system allowing for a near-natural glochidia attachment to host fish that are maintained in tanks fed by the flow-through systems. In some cases, very high F_ST_ values of up to 0.638 between single year cohorts of offspring compared to the original populations were evident (e.g., ST-S with ST-O1). In most cases, these pronounced differences were not consistently present over different year cohorts, especially in captive breeding situations where glochidia from gravid mussels are annually collected in the wild to infest host fish in the rearing facility and where these source mussels differ from year to year.

### Genetic variability

**Figure 2:**
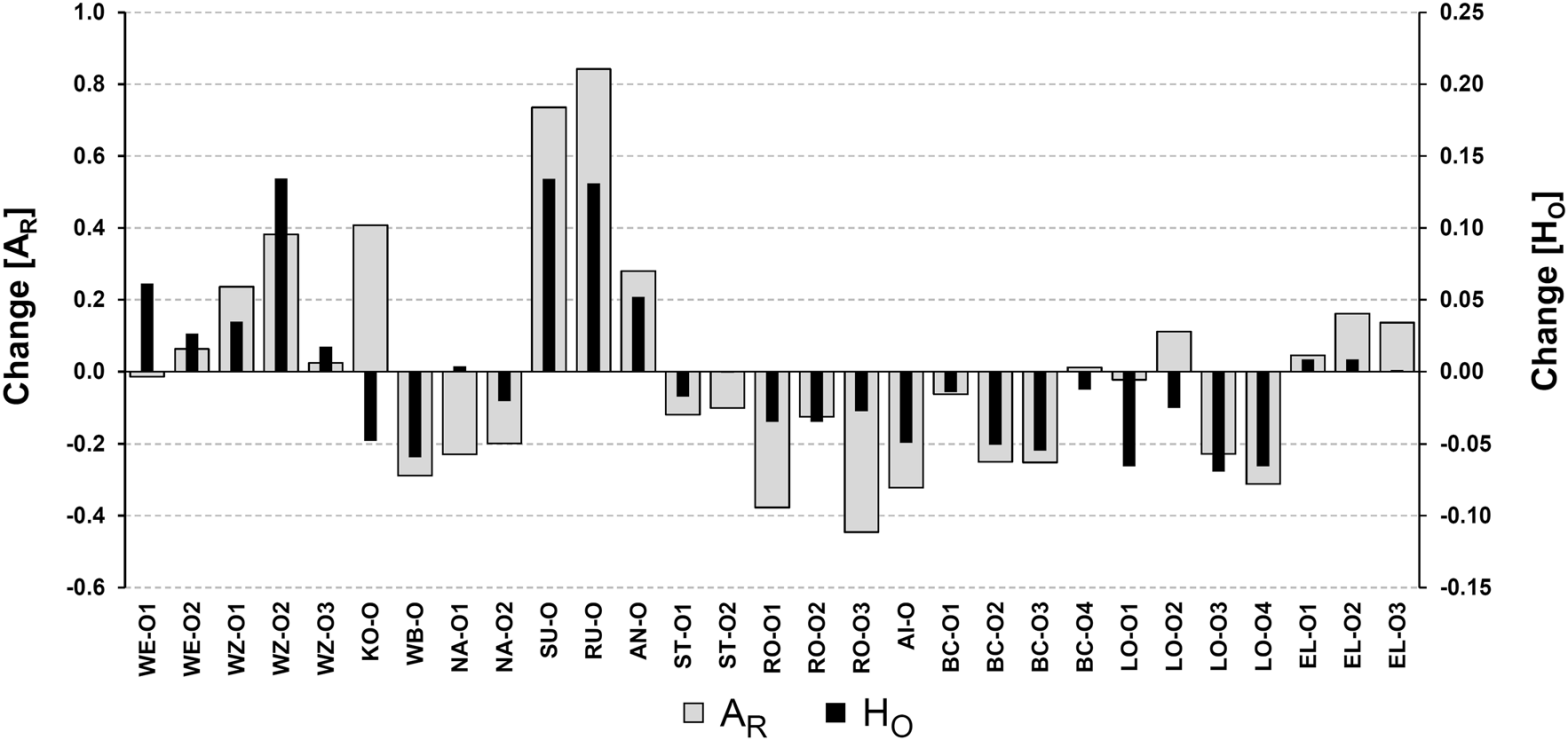
Change of allelic richness (A_R_) and observed heterozygosity (H_O_) of 29 captive bred *Margaritifera margaritifera* cohorts compared to corresponding source populations.

Mean values of observed heterozygosity (H_O_) and allelic richness (A_R_) in source populations ranged from H_O_ = 0.006 (EL) to H_O_ = 0.460 (WB) and from A_R_ = 1.1 (EL and RU) to A_R_ = 2.9 (WB) while the global means of source populations (H_O_ = 0.131, A_R_ = 1.7) were slightly higher than that of juvenile cohorts (H_O_ = 0.112, A_R_ = 1.6). In most cohorts of propagated juveniles, remarkably low levels of these two diversity parameters were detected, but also in the majority of the source populations, especially the westernmost ones (Table 1). However, in comparison to the source populations both A_R_ and H_O_ decreased in over half of the juvenile cohorts (Fig. 2) while respective individual inbreeding coefficients tended to increase (Fig. S1). Another remarkable finding relates to the numbers of physically deformed juveniles. In all six cohorts with ≥ 3 deformed specimens (ST-O1, ST-O2, RO-O1, RO-O2, LO-O2, EL-O1), observed heterozygosities were below 0.05 (Tab. 1).

## Discussion

The value and usefulness of captive breeding in fish populations has been subject to a great controversy^61^ and the increasing number of captive breeding efforts in freshwater mussels^37,38^, particularly in freshwater pearl mussel^11–14,35,36^, makes it necessary to critically reflect on these measures. Our study provides a first analysis of 29 captive-bred annual cohorts of freshwater pearl mussel in comparison to the genetic constitution of populations from their original streams considering four different European countries. Breeding efforts were found to represent a broad, yet not complete spectrum of the genetic diversity of wild populations, and seem to be a valuable tool to at least partially secure the genetic and evolutionary potential of populations at the brink of extinction until efforts of habitat restoration succeed and populations manage to sufficiently recruit in the wild. The findings also illustrate the usefulness of genetic monitoring in captive breeding to identify alterations of the genetic constitution in terms of genetic identity as well as decreased genetic variability.

Most breeding stations are focused on local production of juveniles from nearby populations using local fish stocks. The findings of this study with eleven different genetic clusters represented in those breeding efforts suggest that this approach appears generally useful in considering the overall pronounced degree of genetic differentiation among European populations^16^ as well as the co-evolutionary genetic patterning of freshwater pearl mussel and its hosts^17^. A main challenge lies in the simultaneous rearing of multiple populations and year cohorts in the same rearing facility which always poses a certain risk of mixing different populations. Such confusion was also suspected in one of the rearing facilities in this study and the genetic analyses provided a powerful tool in validating and re-assigning the offspring to the correct source population in the wild. Even though this was a rare incident, it may be generally useful to ensure validation of the integrity of captive-bred mussels before they are being released into the wild, especially in situations when the source population is not yet extinct.

Currently, most breeding stations rely on different ways of collecting parent mussels or larvae from the wild versus completely maintaining a suite of parent mussels together with host fishes. Since the primary objective of those breeding stations is to produce juvenile mussels and since there are many time-critical constraints in terms of finding sufficient numbers of gravid females, handling of glochidia larvae and the infestation of host fish, there is typically no time for a documentation of the exact procedures and methods such as numbers of parent mussels and glochidia collected as well as on individual host fish infestations and other rearing commodities that all may also affect the genetic constitution of the offspring. These constraints, along with the very different genetic background of the pearl mussel source populations considered in this study prevent us from systematically linking certain genetic effects to specific attributes of the respective breeding method. Still, the findings of this study suggest that gathering such information as regularly practiced in many state and federal breeding stations in the US, would be very useful. If this cannot be realized, then at least females should be exchanged annually and progeny from a single female should not be used more than once at each reintroduction locality. Moreover, a realistic evaluation of the effects of any captive breeding on the genetic constitution of populations should not look at single cohorts but rather at the cumulative effects over time. Whilst the findings of this study revealed rather low genetic diversity and signs of inbreeding in some of the annual cohorts, pronounced differences were typically observed when comparing the findings for cohorts from different years. Such differences can be explained by different availabilities of gravid mussels (in some populations only single individuals in certain years) as well as different infestation, metamorphosis and rearing success. Variable levels of multiple paternity as previously observed in this species^62,63^ and the Louisiana Pearlshell, *Margaritifera hembeli*, may also play a role^64^. In any case, the results of this study allow drawing the consistent picture that maintaining rearing activities for specific populations over multiple years is most beneficial since it reduces the risk of genetic bottlenecks, drift and selection effects. This is particularly true for a long-lived species like *Margaritifera margaritifera*, where the reproductive period extends over more than 80 years^65^, allowing multiple generations of different age to jointly reproduce. Still, the extremely low genetic diversity values also observed in most of the source populations for captive breeding suggest that the actual efforts to rescue genetically outstanding populations by captive breeding should begin before an erosion of the genetic variability at small effective population sizes. In cases of populations which are already suffering from loss of genetic diversity, an individual-based selection of specimens for the collection of glochidia is recommended, focusing on individuals which best represent the remaining gene pool of the original population. Also, in any breeding effort it is useful to avoid possible selection and drift effects. In the case of freshwater pearl mussel, it is thus mandatory to use a full and diverse suit of fish hosts and avoid fish strains that only result in metamorphosis of few specimens^4,5,24^. This also includes using older than the commonly utilized 0 + fish which were shown to result in higher numbers of developing postparasitic juveniles under captive breeding situations^25^.

The age and size at which captive-bred juvenile pearl mussels should be released into the wild still remains controversial. The greater capability of adaptation^31^ as well as the lower risk of genetic erosion and die-offs in the rearing facility would suggest an early release. Colmated stream beds and other adverse habitat conditions to which older and larger mussels are much more resistant than young ones, as well as the absence of a decreased genetic variability of older cohorts compared to younger ones in this study (see e.g. BC-O1,2,3,4 and WE-O1, WE-O2 with ages of more than 10 years), along with the continuous improvements of survival in rearing facilities^32,35,36,38,66^ clearly suggest that stocking with such cohorts is also a feasible option.

Effective conservation of freshwater pearl mussel will likely depend on a combination of habitat restoration and effective captive breeding to rescue genetically unique populations over time. An integration of scientific findings into the ecology and conservation genetics of the species is thus likely to be most successful^3^. Given the well-established knowledge on the genetic constitution of freshwater pearl mussel throughout its European and North American distribution^15–23^ and the large and increasing number of breeding facilities for this species, a more systematic approach of evidence-based conservation and restoration^67^ can be recommended. Such an approach should also include systematic selection of priority populations for conservation, effective breeding strategies avoiding effects of in- and outbreeding by genetically informed selection of parent individuals, and a network of collaboration among the different facilities.

## Supplementary Information

Supplementary Information.

## Data Availability

The original data used for the study will be available from the Dryad Digital Repository.
