# Supplementary material for: Securing genetic integrity in freshwater pearl mussel propagation and captive breeding
Source: Sci Rep. 2021 Aug 6;11:16019. doi: 10.1038/s41598-021-95614-2 (PMC8346490; doi:10.1038/s41598-021-95614-2)
Supplement: Supplementary file 1 — Supplementary Information. [file 41598_2021_95614_MOESM1_ESM.docx]

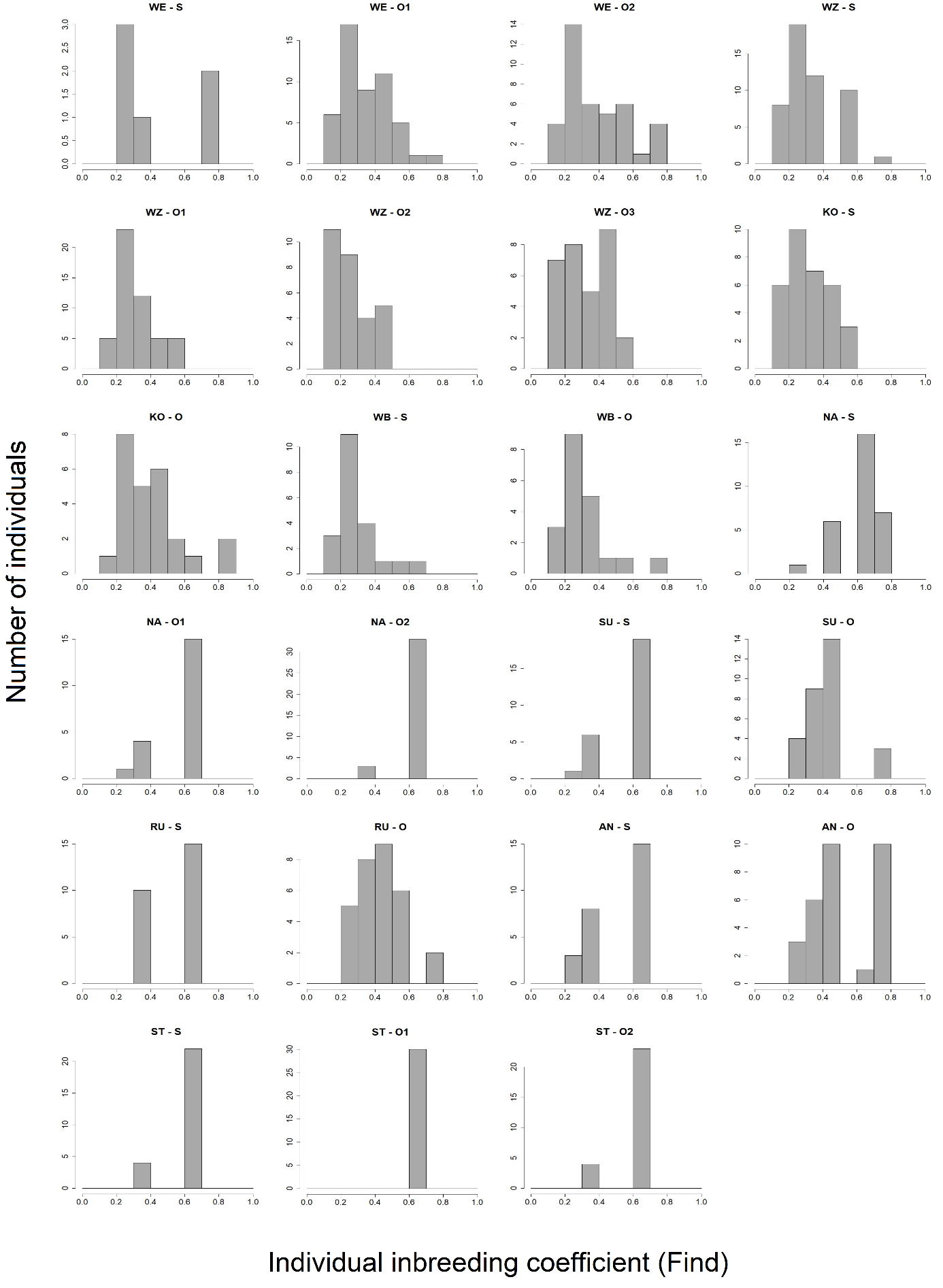


Supplementary Figure S1: Individual inbreeding coefficient (F_ind_) per source population and cohorts of propagated juveniles of *Margaritifera margaritifera*. Population codes according to Table 1.


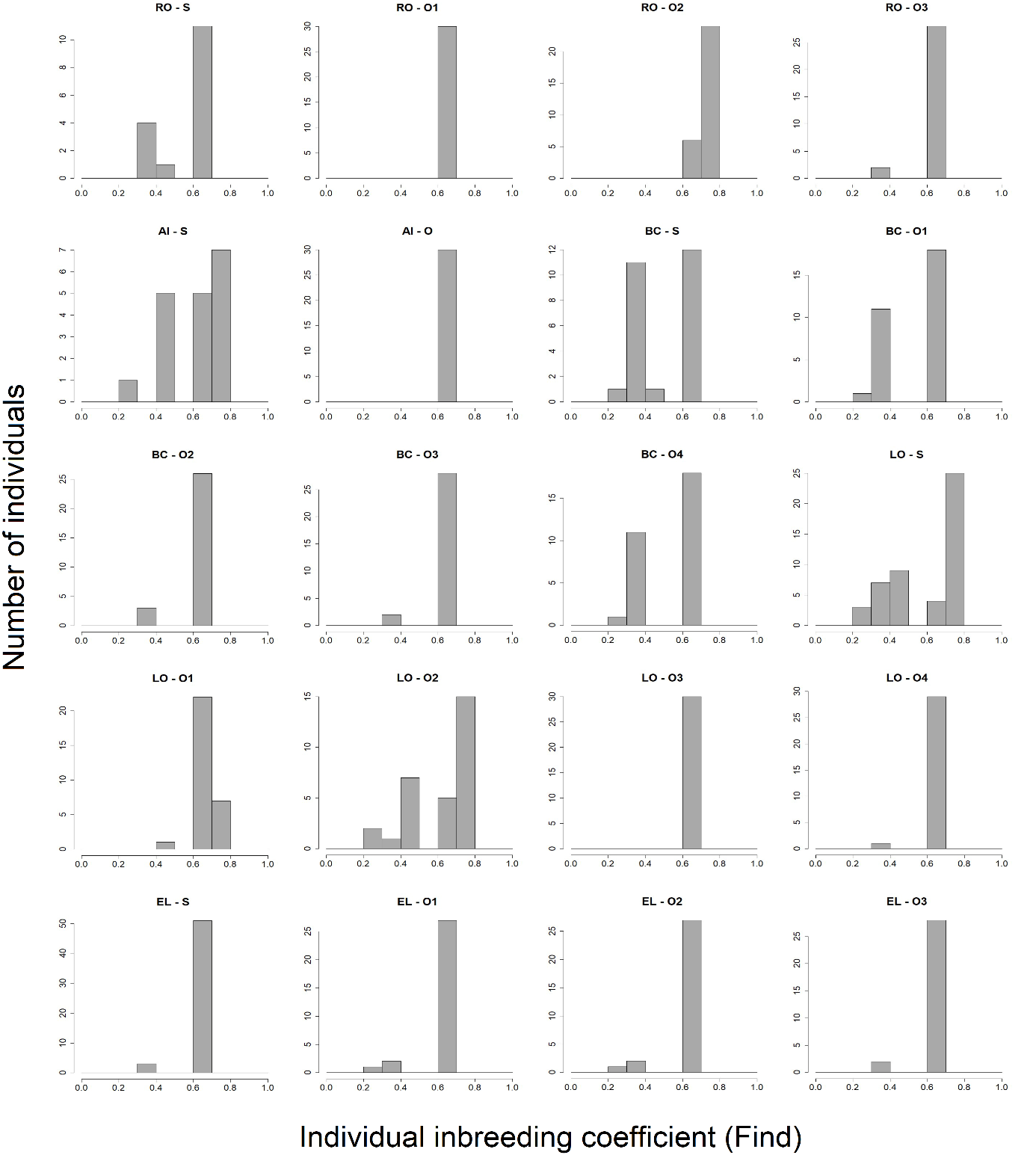


Supplementary Figure S1: continued.


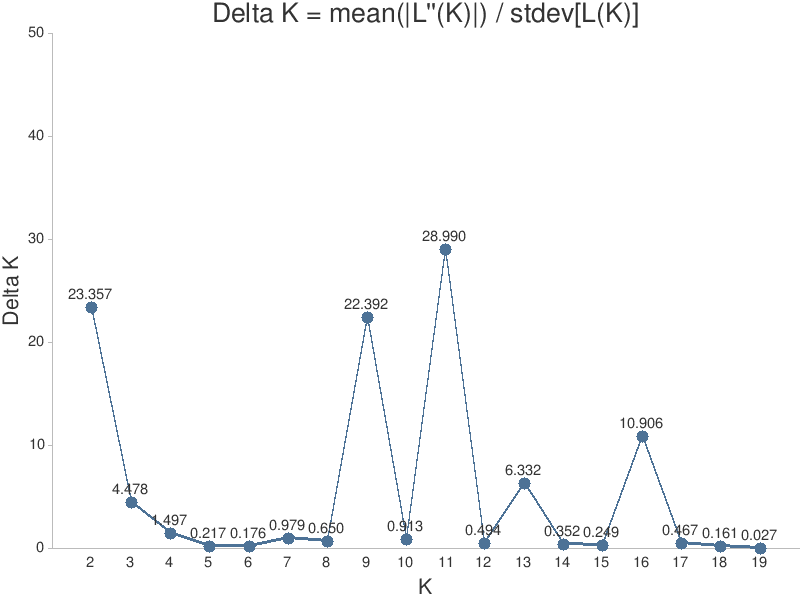


Supplementary Figure S2: Inference of the number of genetic clusters (K) of 1300 *Margaritifera margaritifera* individuals representing 14 source populations and 29 respective cohorts of propagated juveniles from Structure results using the ΔK method.
